# Responsiveness of EORTC QLQ-C30, QLQ-CR38 and FACT-C quality of life questionnaires in patients with colorectal cancer

**DOI:** 10.1186/1477-7525-9-70

**Published:** 2011-08-22

**Authors:** Lionel Uwer, Christine Rotonda, Francis Guillemin, Joëlle Miny, Marie-Christine Kaminsky, Mariette Mercier, Laetitia Tournier-Rangeard, Isabelle Leonard, Philippe Montcuquet, Philippe Rauch, Thierry Conroy

**Affiliations:** 1 Centre Alexis Vautrin, Department of Medical Oncology, Nancy, France; 2 Nancy-University, Paul Verlaine Metz University, Paris Descartes University, EA 4360 Apemac, Nancy, France; 3 INSERM, CIC-EC CIE6, Nancy, France; 4 Quality of Life in oncology platform, Canceropole Grand-Est, Nancy, France; 5 University hospital Jean Minjoz, Department of Radiation Oncology, Besançon, France; 6 Medical and Pharmaceutical University, Department of Biostatistics, Besançon, France; 7 Centre Alexis Vautrin, Department of Radiation Oncology, Nancy, France; 8 Clinique Saint-Vincent, Medical Oncology, Besançon, France; 9 Centre Alexis Vautrin, Department of Surgery, Nancy, France

**Keywords:** Colorectal cancer, Quality of life, EORTC QLQ-C30, EORTC QLQ-CR38, FACT-C, Responsiveness

## Abstract

**Background:**

The aim of this study was to compare the responsiveness of the European Organization for Research and Treatment (EORTC) quality of life questionnaires (QLQ-C30, QLQ-CR38) and the Functional Assessment of Cancer Therapy-colorectal version 4 questionnaire (FACT-C).

**Method:**

This prospective study included 127 patients with colorectal cancer: 71 undergoing chemotherapy and 56 radiation therapy. Responsiveness statistics included the Standardized Response Mean (SRM) and the Effect Size (ES). The patient's overall assessment of his/her change in state of health status was the reference criterion to evaluate the responsiveness of the QoL questionnaires.

**Results:**

34 patients perceived their health as stable and 17 as improved between the first and the fourth courses of chemotherapy. 21 patients perceived their health as stable and 22 as improved between before and the last week of radiotherapy.

The responsiveness of the 3 questionnaires differed according to treatments. The EORTC QLQ-C30 questionnaire was more responsive in patients receiving chemotherapy, particulary functional scales (SRM > 0.55). The QLQ-CR38 and the FACT-C questionnaires provided little clinically relevant information during chemotherapy or radiotherapy.

**Conclusion:**

The EORTC QLQ-C30 questionnaire appears to be more responsive in patients receiving chemotherapy.

## Introduction

Colorectal cancer (CRC) is common in Western societies. The management of locally advanced rectal cancer includes preoperative chemoradiotherapy and surgery. Surgery followed by adjuvant or palliative chemotherapy is the standard of care of localized or advanced stages of CRC. These treatments may affect the patient's quality of life (QoL) and may be responsible for late side-effects or sequelae. QoL assessment is essential to better inform clinical decisions by providing insights into the patient's experiences of disease and treatment [1].

Many questionnaires assess the QoL of patients with CRC. The more frequently used are [2]: the European Organization for Research and Treatment of Cancer (EORTC) QoL QLQ-C30 [3], validated in all European languages [4], the colorectal module QLQ-CR38 [5], and the Functional Assessment of Cancer Therapy-General version FACT-G [6] named FACT-C [7] when specific concerns of patients with CRC are added to common items to all cancer patients. Few data are available in the literature on the responsiveness of these questionnaires [4,8].

Responsiveness, or "sensitivity to change", an essential property of measuring instrument, is defined as the ability to detect a clinically meaningful change [9-12], such as a change that clinicians or patients think is discernable and important. Changes may be spontaneous, due to progression of the disease or may be induced by a therapeutic intervention [13,14]. In a clinical trial, the knowledge of instrument's responsiveness helps in the selection of measures, in setting the correct sample size and assists in prioritising the number of outcomes to be assessed. Responsiveness is the most important property of a questionnaire for use within randomized trials as fewer patients need to be included to demonstrate a significant difference between two treatment options when a more sensitive questionnaire is available [1] would be more accurately detected by disease-specific scales, although they have a narrow focus, have generally been reported to be more responsive than generic health status measures [15]. The EORTC QLQ-CR38 or FACT-C may be more relevant for patients with CRC and their physicians, as they are set to more accurately detect specific clinical effects of the disease. Nevertheless, the selection of instrument for use in a cancer trial should depend on the fit between its content and the objectives of the study [8] and the responsiveness of questionnaires may also vary according to the treatments received.

Both instruments (QLQ-C30 followed by QLQ-CR38 and FACT-C) have subscales measuring physical, emotional, functional, and social aspects; the EORTC QLQ-C30 has additional subscales and single items assessing cognitive function, symptoms, and the financial impact of the disease. The QLQ-CR38 is mainly centered on surgery consequences and has some items about radiotherapy side-effects [16]. The EORTC instrument focuses on the QoL consequences of physical limitations and on clinical symptoms; it may be appropriate for use in clinical trials, while FACT-C emphasises rather satisfaction with daily life [8]. In addition, these questionnaires are different in regard to the phrasing of the items: the EORTC instrument uses questions, and the FACT-C uses statements.

This study was therefore designed to compare the responsiveness of the EORTC QLQ-C30, QLQ-CR38 and FACT-C questionnaires, based on the course of QoL during different treatments for CCR.

## Patients and methods

This is a prospective longitudinal study carried out in four French hospitals (Alexis Vautrin Cancer Centre, Nancy and Oscar Lambret Cancer Centre, Lille; Nancy and Besançon university hospitals) between April 2003 and February 2007. The institutional review board had approved the study.

### Patients

Patients who had histologically proven colorectal adenocarcinoma, an age above 18 years, able to read, speak and write French, no psychological condition that might potentially hamper compliance with QoL assessment, and life expectancy greater than 3 months were eligible.

Patients were not eligible if participating in another QoL survey, in case of cognitive impairment, or previous or concomitant other cancer. Written informed consent and permission from an ethics committee were obtained.

Two different groups of patients were recruited:

- before starting adjuvant or palliative chemotherapy (all fluoropyrimidine based regimens) (chemotherapy group),

- before starting preoperative radiotherapy for rectal cancer (radiotherapy group): Preoperative radiotherapy delivered 45 grays in 25 fractions (five weeks) with or without concomitant fluoropyrimidine.

### Measures and data collection procedure

Patients' characteristics and clinical data were collected at baseline. QoL assessment was performed using three questionnaires: the Functional Assessment of Cancer Therapy-Colorectal (FACT-C version 4.0), the EORTC QLQ-C30 version 3.0 and the EORTC QLQ-CR38. The EORTC colorectal questionnaire (QLQ-CR38) was developed to be used in conjunction with the QLQ-C30. The FACT-C combines the FACT-G with a CRC subscale (CCS). The 3 questionnaires FACT-C, QLQ-C30 and QLQ-CR38 were administrated together. The order in which these questionnaires were administrated to patients was randomized by center from a pre-established table of random number to avoid any systematic order.

According to the type of treatment, different timings of QoL measurements were used:

- Patients treated by adjuvant or palliative chemotherapy were asked to complete all questionnaires (EORTC QLQ-C30, EORTC QLQ-CR38 and FACT-C) before the first, the third and the fourth courses of chemotherapy administered at 2 weeks interval.

- Patients treated by preoperative radiotherapy for rectal cancer were asked to fill the three questionnaires before the first fraction and during the last week of radiotherapy.

The developers of the EORTC QLQ-C30 provide information on what a clinically important difference is for this questionnaire. They have suggested that in groups, the mean change scores was about 5 to 10 in the means scores for those who reported a "little" change, about 10 to 20 for those who reported "moderate" change, and more than 20 for those who reported a large change [17].

The change in health status was assessed between baseline and before the fourth course for patients receiving chemotherapy, and between baseline and the last week of radiotherapy for patients treated with radiation therapy. The patient's overall assessment of his/her change in state of health was the reference criterion to evaluate the responsiveness of the questionnaires. The question was worded: "Compared to the situation before your treatment, how would you evaluate your state of health?" It was recorded according to seven levels of answers: the first 3 answers (much worse, moderately worse and slightly worse) were considered to reflect a clinically significant deterioration, the fourth answer (no change) was considered to reflect stability and the last 3 answers (slightly better, moderately better and much better) were considered to reflect a clinically significant improvement [18].

### Statistical analysis

Statistical analysis was conducted using a pre-specified plan. Completed questionnaires were scored according to the developers' instructions. Transformed FACT-C subscores were determined by converting the original values linearly to a range of 0 (worst QoL) to 100 (best QoL) to facilitate readability of the tables. QoL scores (quantitative variables in each scales) were expressed as mean and standard deviation. The Wilcoxon test was used to test differences between values of the two measurements in each group. Statistical significance for analyses was considered to be at p < 0.05.

Test-retest reliability was assessed between the third and the fourth courses of chemotherapy at two weeks interval in patients who reported no change in their state of health. To assess reliability, a sample of 30 patients with stable health between the 2 testing times was considered as sufficient because it can be approximated by the standard normal distribution. The Intra-class correlation coefficient (ICC) was computed from a one-way analysis of variance (ANOVA). ICC is the ratio of the variance of interest to the sum of the variance of interest plus error [19]. PROC GLM in SAS 9.0. calculated the between and within subject variation, the ICC and its confidence limits. A value between 0 and 0.2 is usually considered to be poor, between 0.2 and 0.4 fair, between 0.4 and 0.6 moderate, between 0.6 and 0.8 good, and a value greater than 0.8 is usually considered to be excellent [20].

Responsiveness statistics included the Standardized Response Mean (SRM) and the Effect Size (ES). It is based on the comparison of signal (mean change in clinically changed patients) to noise (SD of change in stable patients). The SRM is calculated as the mean change in scores between baseline and follow-up divided by the standard deviation (SD) of this change [21]. The ES is used to interpret differences or changes in health related to quality of life following treatment. It is calculated as the mean change in scores between baseline and follow-up divided by the SD of the baseline score [12]. Effect size of 0.2, 0.5 and 0.8 is typically considered as small, moderate and large changes, respectively [20]. We used the same threshold levels for SRM. Cohen's effect size may be influenced by the degree of homogeneity or heterogeneity in the sample. SRM is sensitive to within-subject variability, while ES is sensitive to between-subjects variability. Indeed, the ES statistics relates change over time to the SD of baseline scores and the SRM compares change to the SD of change. Responsiveness was assessed using data from patients deemed to have improved as recommended [22]. We used a bootstrap methods to estimate 95% confidence intervals for the SRM and ES [23]. For this study, it was estimated that a sample at least 30 patients, based on normal distribution assumption should be used.

Data were analysed using Statistical Analysis System software version 9.0 (Cary, North Carolina, USA, 2004) for Windows.

## Results

### Sociodemographic and clinical data

Between April 2003 and February 2007, 127 patients were enrolled in the study: 71 patients in the chemotherapy group and 56 patients in the radiotherapy group. Sociodemographic characteristics of the study population and medical data are presented in Table 1. Compliance with the study was excellent and all patients filled all QoL questionnaires.

**Table 1:** Sociodemographic and clinical patient characteristics

	Chemotherapy Group	Radiotherapy Group	p	Total
**N**	**71**	**56**		**127**

**Age (years)**	60	66	0.001	64
Median				

**Gender (%)**				
Men	38 (53)	43 (77)	0.007	81 (64)
Women	33 (47)	13 (23)		46 (36)

**Marital status (%)**				
Single	3 (4)	4 (7)		7 (6)
Married	53 (69)	44 (80)	0.52	97 (76)
Widower	7 (6)	5 (4)		12 (9)
Divorced	8 (21)	3 (9)		11 (6)

**Localisation (%)**				
Colon	55 (76)	1 (2)	< .0001	56 (44)
Rectum	16 (24)	55 (98)		71 (56)

**Disease status (%)**				
Non metastatic	30 (42)	50 (89)		80 (63)
Metastatic	40 (56)	5 (9)	< .0001	45 (35)
Unknown	1 (2)	1 (2)		2 (2)

Between the first and the fourth courses of chemotherapy, 56 (78.9%) patients answered the question on the perceived change in health: five patients perceived their health as worsened, 34 as stable and 17 as improved.

Between the starting and the last week of radiotherapy, 48 (85.7%) patients answered the question on the change in their health: ten patients perceived their health as worsened, 21 as stable and 22 as improved.

### Test-retest reliability

The test-retest reliability of the questionnaires was studied among 34 patients receiving chemotherapy who perceived their health as stable (Table 2). The ICC of the QLQ-C30 scales ranged from 0.33 for diarrhoea (ICC = 0.33; CI_95% _= [-0.003 to -0.60]) or Global QoL/GHS ICC = 0.33; CI_95% _= [-0.01 to 0.59]) to 0.87 (CI_95% _= [0.76 to 0.93]) for physical function. The reproducibility of nausea/vomiting subscale was fair (ICC = 0.43; CI_95% _= [0.11 to 0.67]). The ICC of QLQ-CR38 questionnaire was good or excellent except for weight loss (ICC = 0.36; CI_95% _= [0.02 to 0.63]). The reproducibility of all of the FACT-C domains was good with ICC greater than 0.60 except for Social/Family Well-Being (ICC = 0.51; CI_95% _= [0.23 to 0.73]).

**Table 2:** Test-retest reliability (ICC) of the QLQ-C30, QLQ-CR38 and FACT-C for patients with stable colorectal cancer undergoing chemotherapy (data obtained between the third and the fourth treatment)

	n	ICC^a^	95% CI^b^
**EORTC QLQ-C30:**			
***Functional scales:***			
Physical function	34	0.87	0.76-0.93
Role function	33	0.80	0.64-0.90
Emotional function	34	0.73	0.52-0.85
Cognitive function	34	0.79	0.62-0.89
Social function	34	0.84	0.71-0.92
Global QoL/GHS	34	0.33	-0.01-0.59
***Symptom scales:***			
Fatigue	34	0.82	0.67-0.90
Pain	34	0.74	0.54-0.86
Nausea and vomiting	34	0.43	0.11-0.67
***Single items ^c ^:***			
Dyspnoea	34	0.63	0.37-0.79
Sleep disturbance	34	0.76	0.58-0.87
Appetite loss	34	0.74	0.54-0.86
Diarrhoea	34	0.33	-0.003-0.60
Constipation	34	0.54	0.25-0.74
Financial impact	34	0.75	0.56-0.87
**EORTC QLQ-CR38:**			
***Functional scales:***			
Body image	32	0.71	0.49-0.85
Future perspective	32	0.76	0.56-0.87
Sexual functioning	31	0.82	0.66-0.91
Sexual enjoyment	11	NA	NA
***Symptom scales:***			
Radiation-induced effects micturition	32	0.67	0.43-0.82
Chemotherapy side effects	32	0.70	0.47-0.84
General gastrointestinal symptoms	32	0.65	0.39-0.81
Defecation problems	26	NA	NA
Stoma-related problems	6	NA	NA
Sexual dysfunction of men	14	NA	NA
Sexual dysfunction of women	3	NA	NA
Weight loss	32	0.36	0.02-0.63
**FACT-C:**			
Physical Well-Being	34	0.76	0.57-0.87
Social/Family Well-Being	33	0.51	0.23-0.73
Emotional Well-Being	34	0.79	0.62-0.89
Functional Well-Being	34	0.73	0.52-0.85
Colorectal Cancer Specific	34	0.77	0.59-0.90
FACT-C total score	34	0.75	0.55-0.87
Trial Outcome Index-Colorectal	34	0.82	0.66-0.90

^a ^Intraclass correlation coefficient; ^b ^confidence interval; NA: Not Applicable (n < 30)

### Description of the QoL scores and responsiveness of the questionnaires

#### - Chemotherapy group

Because only 5 patients perceived their health as worsened, the responsiveness has been studied only in patients with improvement of their health. Between the first and the fourth courses of chemotherapy, 17 patients perceived their health as improved: 12 patients were slightly better, 3 moderately better and only 2 patients were much better. All patients except two had previous surgery for resection of the primary tumor before chemotherapy. Seven patients received adjuvant chemotherapy and 10 patients were treated for metastatic disease. In these patients with improved health status, no difference in changes of scores between baseline and before the fourth course of chemotherapy has been found between patients with adjuvant treatment versus those treated for metastatic disease (data not shown).

##### Description of scores (Table 3)

**Table 3:** Mean scores before the first course of chemotherapy, changes of scores between the first and the fourth courses of responsiveness statistics in patients who perceived improved health (n = 17)

	n	Score before 1^st^chemother.Mean (SD ^a^)	Difference Mean (SD ^a^)	P^b^	SRM^c ^(CI 95%)	ES^d ^(CI 95%)
**EORTC QLQ-C30:**						
**Functional scales^e ^:**						
Physical function	16	67.8 (30.2)	+14.9 (22.5)	**0.02**	0.64 (0.19;1.08)	0.48 (0.14;0.86)
Role function	16	55.9 (39.1)	+21.9 (16.7)	**0.03**	0.60 (0.08;1.13)	0.56 (0.10;1.07)
Emotional function	17	69.6 (16.1)	+16.2 (16.7)	**0.003**	0.84 (0.28;1.54)	1.00 (0.35;1.68)
Cognitive function	17	77.5 (20.4)	+10.8 (16.6)	**0.02**	0.64 (0.16;1.29)	0.52 (0.14;1.09)
Social function	17	62.7 (34.1)	+13.7 (38.3)	0.16	0.35 (-0.17;0.88)	0.40 (-0.20;0.89)
Global QoL/GHS	17	54.9 (20)	+14.2 (14.7)	**0.001**	0.96 (0.38;1.67)	0.71 (-0.23;1.44)
**Symptom scales^f ^:**						
Fatigue	16	42.8 (27.9)	-16.3 (25.8)	**0.02**	- 0.63 (-1.07;-0.19)	- 0.58 (-1.13;-0.15)
Pain	17	28.4 (31)	-17.6 (37.5)	0.07	- 0.47 (-0.90;-0.09)	- 0.56 (-1.07;-0.05)
Nausea and vomiting	16	12.7 (22.5)	-6.2 (21.8)	0.27	- 0.28 (-0.69;0.28)	- 0.27 (-0.66;0.25)
**Single items^f ^:**						
Dyspnoea	16	21.6 (26.2)	-8.3 (25.8)	0.22	- 0.32 (-1.05;0.65)	- 0.31 (-0.89;0.17)
Sleep disturbance	16	45.1 (31)	-12.5 (38.2)	0.21	- 0.32 (-0.86;0.19)	- 0.40 (-0.98;0.38)
Appetite loss	16	29.4 (30.9)	-12.5 (26.9)	0.08	- 0.46 (-0.93;0.01)	- 0.40 (-0.87;0.08)
Diarrhoea	17	19.6 (23.7)	+3.9 (33.1)	0.63	0.11 (-0.38;0.64)	0.16 (-0.41;1.17)
Constipation	17	17.6 (29.1)	-5.9 (27.0)	0.38	- 0.21 (-0.75;0.31)	- 0.20 (-0.67;0.31)
Financial impact	17	5.9 (17.6)	+3.9 (11.1)	0.16	0.35 (0.11;0.57)	0.22 (-0.05;0.49)

**EORTC QLQ-CR38:**						
**Functional scales^e^:**						
Body image	16	81.7 (28)	+3.8 (14.2)	0.30	0.27 (-0.33;0.69)	0.13 (-0.13;0.37)
Future perspective	16	45.1 (28.7)	+22.9 (31.5)	**0.01**	0.70 (0.27;1.27)	0.79 (0.27;1.38)
Sexual functioning	14	20.2 (20.9)	+5.9 (16.8)	0.21	0.37 (-0.28;0.86)	0.28 (-0.15;0.83)
Sexual enjoyment	7	54.2 (43.4)	+9.5 (41.8)	0.57	0.23 (-0.56;1.28)	0.21 (-0.73;0.91)
**Symptom scales^f ^:**						
Radiation-induced effects micturition	16	23.5 (15.2)	+4.8 (15.7)	0.23	0.30 (-0.23;0.91)	0.32 (-0.23;0.89)
Chemotherapy side effects	16	18.6 (19.5)	+4.2 (18.4)	0.38	0.23 (-0.30;1.1)	0.21 (-0.77;0.92)
General Gastrointestinal symptoms	16	23.0 (12)	-7.0 (14.3)	0.07	-0.49 (-1.38;0.11)	- 0.58 (-1.45;0.17)
Defecation problems	13	15.4 (11.5)	-1.8 (8.2)	0.43	-0.22 (-1.10;0.41)	- 0.16 (-0.65;0.32)
Stoma-related problems	4	31.0 (21.5)	-1.2 (13.7)	0.87	-0.10 (-1.15;1.02)	- 0.05 (-1.96;2.43)
Sexual dysfunction of men	8	43.8 (41.7)	-2.1 (35.0)	0.87	-0.06 (-1.43;0.82)	- 0.04 (0.84;0.64)
Sexual dysfunction of women	2	8.3 (11.8)	-8.3 (11.8)	0.50	-0.70 (-1.44;-0.31)	-0.70 (-1.44;-0.31)
Weight loss	16	25.5 (34.4)	-22.9 (33.8)	**0.02**	-0.67 (-1.01;-0.35)	- 0.66 (-1.01;-0.36)

**FACT-C^e^:**						
Physical Well-Being	17	78.0 (15.3)	+5.3 (15.8)	0.18	0.34 (-0.20;0.85)	0.34 (-0.17;0.87)
Social/Family Well-Being	17	74.3 (21.1)	+3.2 (18.3)	0.48	0.17 (0.47;0.59)	0.15 (-0.39;0.48)
Emotional Well-Being	17	74.3 (19.6)	+3.3 (13.7)	0.34	0.24 (-0.32;0.64)	0.16 (-0.16;0.48)
Functional Well-Being	17	50.8 (17.1)	+10.0 (13.2)	**0.007**	0.75 (0.20;1.27)	0.58 (0.19;0.93)
Colorectal Cancer Specific	16	69.6 (10.6)	+3.9 (12.9)	0.25	0.30 (-0.26;0.88)	0.36 (-0.27;0.98)
FACT-C total score	17	69.5 (8.6)	+1.6 (9.0)	0.49	0.19 (-0.36;0.71)	0.18 (-0.35;0.81)
Trial Outcome Index-Colorectal	17	66.4 (10.6)	+4.7 (13.0)	0.152	0.36 (-0.20;0.96)	0.44 (-0.29;0.99)

^a ^Standard Deviation. ^b ^Wilcoxon test for the difference. ^c ^Standardized Response Mean. ^d ^Effect Size

^e ^Higher score indicates a higher level of functioning or better quality of life. ^f ^Higher score indicates more symptoms/problems. Bold indicate p values < 0.05.

Between the first and the fourth courses of chemotherapy, patients reported a "moderate" change for 5 functional scales (difference of scores greater than 10 points) and they reported a large change for the role function scale (difference of scores greater than 20 points). All these scales had a statistically significant (p < 0.05) except for the "social function" scale.

Patients also reported a "moderate" change of fatigue, pain, insomnia and appetite loss with a decrease of these symptoms (difference of scores greater than 10 points). But only the "fatigue" domain had a statistically significant (Δ = -16.3 (SD = 25.8), p = 0.02).

Considering the QLQ-CR38 scores, a statistically significant difference of the scores was only observed for the future perspective single item and the weight loss scale which showed "very much" change with a difference scores greater than 20 points (Δ_future perspective _= +22.9 (SD = 31.5), p = 0.01 and Δ_weight loss _= -22.9 (SD = 33.8), p = 0.02).

For the FACT-C scales, the only statistically significant difference of the scores was observed for the Functional Well-Being subscale (Δ = +10.0 (SD = 13.2), p = 0.007).

##### Responsiveness (Table 3)

The indicators of responsiveness (SRM and ES) have been calculated for patients with improved health. The physical, role, emotional and cognitive function and the fatigue scale of the QLQ-C30 appeared to be responsive with values of the indicators (SRM and ES) greater than 0.5 reflecting moderate ability to detect an effect of chemotherapy treatment. The SRM for the global QoL/GHS score reflected moderate ability to detect treatment effect (SRM = 0.96). The indicators of responsiveness for the future perspective, weight loss and sexual dysfunction in women subscales of the QLQ-CR38 questionnaire reflected moderate ability to detect an effect of chemotherapy on change of clinical state (absolute values of SRM and ES between 0.51 and 0.79).

The changes in clinical state evaluated by the total score of the FACT-C questionnaire were not significant (SRM and ES < 0.20). Only a moderate ability of the Functional Well-Being scale to detect an effect of treatment was observed (SRM and ES between 0.58 and 0.75).

#### - Radiotherapy group

Between before radiotherapy and the last week of treatment, ten patients perceived their health as worsened, 21 as stable and 22 as improved. Among 22 patients with improvement, 16 patients were slightly better, 4 moderately better and only 2 patients were much better.

##### Description of scores (Table 4)

**Table 4:** Mean scores before the radiotherapy, change of scores between before and the last week of radiotherapy and responsiveness statistics in patients who perceived improved health (n = 22)

	n	Score before radiotherapy Mean (SD^a^)	Difference Mean (SD^a^)	P^b^	SRM^c ^(CI 95%)	ES^d ^(CI 95%)
**EORTC QLQ-C30:**						
**Functional scales^e ^:**						
Physical function	22	83.9 (18.4)	-6.5 (16.4)	0.08	- 0.39 (-0.78;0.02)	- 0.35 (-0.85;0.21)
Role function	21	74.6 (27.2)	-6.3 (28.1)	0.07	- 0.22 (-0.74;0.22)	-0.23 (-0.78;0.21)
Emotional function	22	79.2 (17.8)	+3.8 (14.2)	0.23	0.26 (-0.19;0.67)	0.21 (-0.15;0.57)
Cognitive function	22	94.7 (7.9)	-4.5 (15.6)	0.19	- 0.29 (-0.67;0.17)	- 0.57 (-0.55;0.21)
Social function	22	78.8 (24.2)	-5.3 (22.0)	0.27	- 0.24 (-0.69;0.21)	- 0.21 (-0.78;0.16)
Global QoL/GHS	22	54.9 (22.7)	+5.7 (26.4)	0.32	0.21 (-0.32;0.53)	0.25 (-0.24;0.75)
**Symptom scales^f ^:**						
Fatigue	22	38.9 (25.1)	+2.0 (28.4)	0.74	0.07 (-0.37;0.56)	0.08 (-0.37;0.70)
Pain	22	23.5 (23.9)	+8.3 (27.6)	0.17	0.30 (-0.16;0.84)	0.34 (-0.16;1.04)
Nausea and vomiting	22	2.3 (7.8)	+3.0 (12.2)	0.26	0.24 (-0.21;0.70)	0.38 (-0.75;1.33)
**Single items^f ^:**						
Dyspnoea	21	11.1 (16.1)	0.0 (14.9)	1.00	0.0 (-0.52;0.44)	0.0 (-0.41;0.38)
Sleep disturbance	22	28.8 (34.6)	-7.6 (35.5)	0.33	- 0.21 (-0.62;0.30)	- 0.21 (-0.64;0.30)
Appetite loss	21	20.6 (28.8)	+4.8 (35.4)	0.54	0.13 (-0.31;0.66)	0.16 (-0.32;0.91)
Diarrhoea	22	28.8 (31.4)	-1.5 (33.3)	0.83	- 0.04 (-0.56;0.39)	- 0.04 (-0.48;0.56)
Constipation	22	15.2 (24.6)	+12.1 (24.2)	**0.03**	0.50 (0.06;0.97)	0.49 (0.06;1.20)
Financial impact	21	10.6 (26.0)	+5.7 (26.4)	0.33	0.22 (0.17;0.31)	0.06 (-0.15;0.24)

**EORTC QLQ-CR38:**						
**Functional scales^e ^:**						
Body image	20	90.9 (16.3)	-3.3 (14.5)	0.32	- 0.23 (-0.7;0.27)	- 0.20 (-0.81;0.50)
Future perspective	21	56.1 (26.0)	+4.8 (21.8)	0.33	0.21 (-0.23;0.72)	0.18 (-0.21;0.57)
Sexual functioning	20	16.7 (27.9)	-5.8 (21.8)	0.24	- 0.26 (-0.76;0.19)	- 0.20 (-0.54;0.17)
Sexual enjoyment	10	33.3 (49.4)	-26.7 (30.6)	**0.02**	- 0.87 (-1.79;-0.45)	- 0.53 (-1.03;-0.15)
**Symptom scales^f ^:**						
Radiation-induced effects micturition	21	21.7 (22.6)	+19.3 (21.9)	**0.0006**	0.88 (0.42;1.44)	0.85 (0.36;1.50)
Chemotherapy side effects	21	15.2 (23.6)	+5.3 (13.0)	0.08	0.40 (-0.11;0.89)	0.22 (-0.15;0.55)
General Gastrointestinal symptoms	21	26.7 (14.9)	-0.4 (11.9)	0.88	- 0.03 (-0.50;0.46)	0.02 (-0.38;0.36)
Defecation problems	19	37.6 (21.6)	-1.5 (14.8)	0.66	- 0.10 (-0.59;0.41)	- 0.07 (-0.42;0.27)
Stoma-related problems	1	9.5 (00.0)	+23.8 (-)	-	-	-
Sexual dysfunction of men	9	35.9 (44.5)	+7.4 (20.6)	0.31	0.35 (-0.28;1.09)	0.16 (-0.09;0.65)
Sexual dysfunction of women	2	16.7 (23.6)	+16.7 (0.0)	1.00	0	0.70 (0.65;1.10)
Weight loss	21	28.8 (34.6)	+1.6 (38.7)	0.85	0.04 (-0.40;0.55)	0.04 (-0.43;0.67)

**FACT-C^e ^:**						
Physical Well-Being	19	81.4 (16.4)	-5.0 (14.3)	0.15	- 0.34 (-0.77;0.17)	- 0.30 (-0.94;0.10)
Social/Family Well-Being	19	71.3 (25.7)	-7.4 (16.2)	0.06	- 0.45 (-0.96;0.09)	- 0.28 (-0.63;0.03)
Emotional Well-Being	19	77.5 (17.6)	0 (12.7)	1.00	0.0 (-0.78;0.31)	0.0 (-0.34;0.37)
Functional Well-Being	18	50.1 (20.0)	-2.5 (17.1)	0.55	- 0.14 (-0.71;0.40)	- 0.12 (-0.56;0.35)
Colorectal Cancer Specific	19	67.7 (11.5)	-3.2 (12.3)	0.27	- 0.26 (-0.64;0.24)	- 0.28 (-0.80;0.18)
FACT-C total score	19	69.1 (11.4)	-3.0 (8.5)	0.14	- 0.35 (-0.96;0.19)	- 0.26 (-0.64;0.15)
Trial Outcome Index-Colorectal	19	65.7 (13.7)	-2.6 (10.2)	0.29	- 0.25 (-0.78;0.31)	- 0.18 (-0.60;0.20)

^a ^Standard Deviation. ^b ^Wilcoxon test of the change. ^c ^Standardized Response Mean. ^d ^Effect Size.

^e ^Higher score indicates a higher level of functioning or better quality of life. ^f ^Higher score indicates more symptoms/problems. Bold indicate p values < 0.05.

A "small" deterioration of physical (Δ = -6.5, SD = 16.4), role (Δ = -6.3, SD = 28.1), and social functioning (Δ = -5.3, SD = 22.0) of QLQ-C30 and a "moderate" increase of constipation (Δ = +12.1, SD = 24.2) was observed during the last week of radiotherapy as compared to baseline; these differences were statistically significant only for constipation (p = 0.03)

No statistically significant changes were observed in QLQ-CR38 scales except for radiation-induced effects micturition scale (Δ = +19.3 (SD = 21.9), p = 0.0006) and sexual enjoyment scale (Δ = -26.7 (SD = 30.6), p = 0.02). Indeed, patients reported a large decrease in sexual enjoyment scores. For the FACT-C scales, no statistically significant differences of the scores including the global score were observed.

##### Responsiveness (Table 4)

The indicators of responsiveness (SRM and ES) have been calculated for patients who perceived their health as improved. The SRM for the global QoL/GHS score was 0.21, reflecting a minimal ability to detect an effect of radiotherapy treatment on clinical change, as well as the pain (SRM = 0.30) and constipation (SRM = 0.50) subscales or items.

Indicators of responsiveness for the various scores of the QLQ-CR38 questionnaire reflected a fair ability to detect a treatment effect on clinical change. Only indicators for radiation-induced effects on micturition and sexual enjoyment (absolute values greater than or equal to 0.71) reflected a good responsiveness.

Analysis of responsiveness in all domains and the global score of the FACT-C questionnaire proved a fair ability to detect a particularly treatment effect. (absolute values of SRM and ES indices between 0.14 and 0.45).

## Discussion

Test-retest reliability and responsiveness are two essential properties of a measuring instrument. To be responsive, a questionnaire should be reproducible [24,25]: if an instrument is unreliable, it will be less responsive.

In CRC patients, the reproducibility of the EORTC QLQ-C30 questionnaires is good or excellent except for nausea/vomiting subscale (which can be explained by changes in the symptom intensity and changes in antiemetic treatment) and surprisingly for QoL/GHS questionnaire. The reproducibility of QLQ-CR38 questionnaire was good or excellent. The FACT-C showed a good reproducibility except for the Social/Family Well-Being subscale (ICC < 0.60). The responsiveness of each of these 3 questionnaires, according to the patient's assessment of his/her change in their state of health, differs according to treatment types: the EORTC QLQ-C30 questionnaire, compared to the QLQ-CR38 and FACT-C, appears as the most appropriate instrument to measure and to detect an effect of chemotherapy on QoL changes. Indicators of responsiveness of the global QoL/GHS score reflect moderate ability (ES) or a good ability (SRM) to detect an effect of chemotherapy on change of health status. In our study, the QLQ-CR38 and the FACT-C questionnaires provided little clinically relevant information during chemotherapy or radiotherapy. Four of the 12 scores of the QLQ-CR38 are related to sexuality and many answers to questions on this topic were missing. These conclusions are similar whatever the indicator of responsiveness used: Standardized Response Mean and Effect Size, all results going in the same direction.

Sensitivity of these questionnaires to detect change compared to the patient's assessment significantly differs according to the type of treatment (chemotherapy or radiotherapy). The best correlation between QoL assessment by self-rating questionnaires and the patient's overall assessment of his/her change in state of health is observed in patients receiving chemotherapy but only with the QLQ-C30. Indeed, measurements of functional scales and global QoL scores of the QLQ-C30 questionnaire are the most sensitive except for social function scale.

Our results suggest a lack of sensitivity of these QoL questionnaires compared to the patient's overall assessment of his/her change in state of health. These results of responsiveness in patients receiving chemotherapy are confirmed by the evolution of the QoL scores between the first and the fourth course of chemotherapy. Indeed, a clinical relevant variation was observed for all functional scales by the QLQ-C30, with mean difference superior to 10 points in ten scales and superior to 5 points for all scales.

These results can be explained by items on chemotherapy side-effects such as fatigue. No significant score's variations appeared with the FACT-C questionnaire which could be because this scale summates individual items that may not be changing in the same direction. Only the Functional Well-Being subscale reflects a good ability to detect an effect of chemotherapy on change of health status in patients.

That is likely to explain that this questionnaire is less responsive than the EORTC QLQ-C30.

It should be noted that, as SRM is unrelated to sample size and unit of measure, it lends itself to comparison between different measures which have been tested on samples of different sizes.

Interpretation of the results of the course of QoL scores and their responsiveness for patients receiving chemotherapy was performed independently of the chemotherapy regimen and of the disease status.

The EORTC QLQ-C30 detect QoL impairments in dimensions that are not specifically related to the primary cancer [26] and to the specific treatment. Consequently, for patients treated by radiotherapy with specific consequences, the indicators of responsiveness of the EORTC QLQ-C30 are interpreted as poor for the most part of functional or symptom scales and single items. The specific consequences of rectal radiotherapy are more accurately detected by adapted disease-specific subscales. The CCR Specific, the TOI Colorectal subscales and the FACT-C total score have been shown to be more responsive than the four general domains in this study such as previously shown by Ward et al [7]. Yost et al [27] identified the minimal important difference (MID): 1-2 points for the CCR specific, 4-6 points for the TOI colorectal and 5-8 points for the FACT-C, original 0-28, 0-84 and 0-136 scale respectively.

However, we did not observe these results for these 3 subscales in patients ongoing radiotherapy.

The FACT-G questionnaire seems particularly useful as it provides a global score making it easier to use in the context of therapeutic trials [15]. It includes only four questions on symptoms and it emphasises rather satisfaction with daily life. It may investigate psychological, social and familial well-being more thoroughly than the EORTC QLQ-C30. The colorectal module (FACT-C) is simple and comprises only nine questions. However, in this study, assessment of QoL by FACT-C questionnaire and its responsiveness are not conclusive. This result suggests the non-relevance of CCR Specific subscale of the FACT-C.

These 3 questionnaires referred to QoL issues are structured differently. Indeed, the QLQ-CR38 contains more symptoms items. The FACT-C questionnaire investigates more functional domains like QLQ-C30. The two specific questionnaires (QLQ-CR38 and FACT-C) have been developed for measuring the same disease so they might be comparable. Nevertheless, only few items are similar (for example: questions about body image and stoma).

Recently an updated version of the QLQ-CR38, the QLQ-CR29, has been developed [28,29]. It has revised scales about sexual functioning and gastrointestinal function to improve participation and compliance. Indeed, the sexual domain is limited to 2 items and this questionnaire separates items for patients with and without stoma.

A limitation of this study is that the responsiveness analysis relied only on patients who perceived their health as improved. We did not analyse the responsiveness in patients with a decline in health due to the low number of patients. The response-shift phenomenon can be a possible explanation for the large percentage of patients who reported an improvement in their health status, while only a small percentage of participants reported a decline in health status. Response-shift can be considered the result of an adaptive response to a changed health status, and as such is viewed as a positive phenomenon. Several studies showed that it is plausible that a change in health perception is not primarily introduced by an intervention (e.g. chemotherapy), but by coping with the disease itself [30,31].

Another limitation is that the questionnaire scales are differently constructed, therefore, it was difficult to compare directly the responsiveness of these questionnaires. Indeed, while some QoL measures include single symptoms items, others include summated scales. For example "diarrhea" is measured by a single item in QLQ-C30 questionnaire. However, diarrhea is part of the "colorectal cancer specific" concerns in FACT-C. Nausea and pain are separate two-item symptom scales in QLQ-C30, while they are both included in the "physical well- being" scale of FACT-C.

We studied the responsiveness during treatment but long-term complications have not been examined. It could be considered a limitation of this study. A strength of the present study is, however, that it was performed in various clinical situations for CRC patients.

## Conclusion

Implications for practice: The present results showed that the responsiveness of QoL questionnaires, an essential property, is different according to the type of treatment. We hypothesized that disease-specific scales tend to be more responsive than generic health status measures. Unfortunately, our results were not conclusive. We observed that EORTC QLQ-C30 functional subscales may be responsive to improvement in overall health state in patients undergoing chemotherapy and we confirm that QLQ-CR38 needs improvement. An updated version of the QLQ-CR38, the QLQ-CR29 is now available [28,29]. Generic instruments provide a broader context in which to interpret the information about change in QoL.

This study on responsiveness of the questionnaires provides arguments for the choice of generic to assess QoL in patients with CRC.

Implications for future research: Further investigation of the responsiveness to change of the EORTC CR29 module is warranted.

## Abbreviations

CRC: ColoRectal Cancer; EORTC: European Organization for Research and Treatment of Cancer; ES: Effect Size; FACT-C: Functional Assessment of Cancer Therapy-Colorectal; FACT-G: Functional Assessment of Cancer Therapy-General; GHS: Global Health Status; QLQ-CR38: Quality-of-Life ColoRectal module; QoL: Quality of Life; RE: Relative Efficiency; SRM: Standardized Response Mean; TOI-C: Trial Outcome Index of FACT-C.

## Competing interests

The authors declare that they have no competing interests.

## Authors' contributions

Conception and design: FG, TC, MM

Provision of study materials or patients: LU, JM, MCK, MM, LT, IL, PM, PR, TC

Collection and assembly of data: LU, CR, IL

Data analysis and interpretation: LU, CR, FG, TC

Manuscript writing: LU, CR, TC, FG

Final approval of manuscript: All authors contributed to the manuscript and have read and approved its final version.
